# Exploring preferences for symptom management in primary care: a discrete choice experiment using a questionnaire survey

**DOI:** 10.3399/bjgp15X685705

**Published:** 2015-06-29

**Authors:** Anne McAteer, Deokhee Yi, Verity Watson, Patricia Norwood, Mandy Ryan, Philip C Hannaford, Alison M Elliott

**Affiliations:** Academic Primary Care; Cicely Saunders Institute of Palliative Care and Rehabilitation, King’s College London, London.; Health Economics Research Unit, Division of Applied Health Sciences, University of Aberdeen, Aberdeen.; Health Economics Research Unit, Division of Applied Health Sciences, University of Aberdeen, Aberdeen.; Health Economics Research Unit, Division of Applied Health Sciences, University of Aberdeen, Aberdeen.; NHS Grampian chair of primary care and vice principal, Academic Primary Care;; Academic Primary Care;

**Keywords:** discrete choice experiment, health services research, primary health care, signs and symptoms, symptom management

## Abstract

**Background:**

Symptoms are important drivers for the use of primary care services. Strategies aimed at shifting the focus away from the GP have broadened the range of primary healthcare available.

**Aim:**

To explore preferences for managing symptoms and investigate trade-offs that the public are willing to make when deciding between different primary care services.

**Design and setting:**

UK-wide postal questionnaire survey of 1370 adults.

**Method:**

A discrete choice experiment examined management preferences for three symptoms of differing seriousness (diarrhoea, dizziness, and chest pain). Willingness-to-pay estimates compared preferences between symptoms, and by sex, age, and income.

**Results:**

Preferences differed significantly between symptoms. ‘Self-care’ was the preferred action for diarrhoea and ‘consulting a GP’ for dizziness and chest pain. ‘Waiting time’ and ‘chance of a satisfactory outcome’ were important factors for all three symptoms, although their relative importance differed. Broadly, people were more prepared to wait longer and less prepared to trade a good chance of a satisfactory outcome for symptoms rated as more serious. Generally, preferences within subgroups followed similar patterns as for the whole sample, although there were differences in the relative strength of preferences.

**Conclusion:**

Despite increased choices in primary care, ‘traditional’ actions of ‘self-care’ for minor symptoms and ‘GP consultation’ for more serious symptoms were preferred. The present findings suggest, however, that people may be willing to trade between different health services, particularly for less serious symptoms. Understanding the relative importance of different factors may help inform interventions aimed at changing management behaviour or improving services.

## INTRODUCTION

Symptoms are common and important drivers of primary care use.^1^,^2^ UK government policy supports strategies that shift some primary care provision away from the GP.^3^ Policies include developing other primary care team members’ roles (for example practice nurses and pharmacists); introducing additional services (for example, nurse-led telephone advice lines); and encouraging self-care for minor symptoms.^4^ Such policies encourage individuals to consider trade-offs, for example, trading between convenience (such as taking over-the-counter medicines), and professional advice (such as consulting a GP). Identifying the trade-offs people are willing to make can inform targeted interventions to influence consultation behaviour, thus informing policy on how primary care services could be configured to meet patient needs while encouraging efficient use of healthcare resources.

This study reports the findings of a discrete choice experiment (DCE) investigating the public’s preferences for managing three symptoms of differing seriousness (diarrhoea, dizziness, and chest pain) in primary care.

## METHOD

### Overview of DCE

A DCE is an economic method used to assess preferences^5^ and is based on the premise that a service can be described in terms of attributes (for example health professional seen) and levels (for example, GP or practice nurse). A DCE can be used to assess whether attributes are important; trade-offs between attributes; and, if cost is included as an attribute, willingness to pay (WTP) — a monetary measure of benefit for changes in attributes and different management configurations.

This DCE was the final phase of a community-based study examining prevalence^6^ and management^7^ of 25 symptoms. Attributes and levels, shown in Appendix 1, were informed by earlier study phases.^8^ Briefly, a symptom survey asked responders to rate the importance of a range of factors that could influence management choices, and semi-structured telephone interviews with a sample of survey responders explored decision-making processes, identifying other factors influencing management choices. A cost attribute was included, with levels informed by the pilot study.

To explore preference heterogeneity across symptom seriousness, responders completed the DCE for three symptom scenarios developed by clinicians to reflect scenarios of increasing seriousness: diarrhoea (non-serious), dizziness (intermediate), and chest pain (serious scenario) (Appendix 2). DCE responders rated the seriousness of each scenario on a scale of 0 (not serious) to 10 (very serious).

**How this fits in**

Increased choice in primary care services encourages individuals to make choices and trade-offs between one type of care and another. However, it is not clear how the public is responding to these choices. The present study explores preferences for symptom management. The results will inform development of interventions aimed at changing behaviour within primary care and encouraging efficient use of healthcare resources.

**Figure 1.**
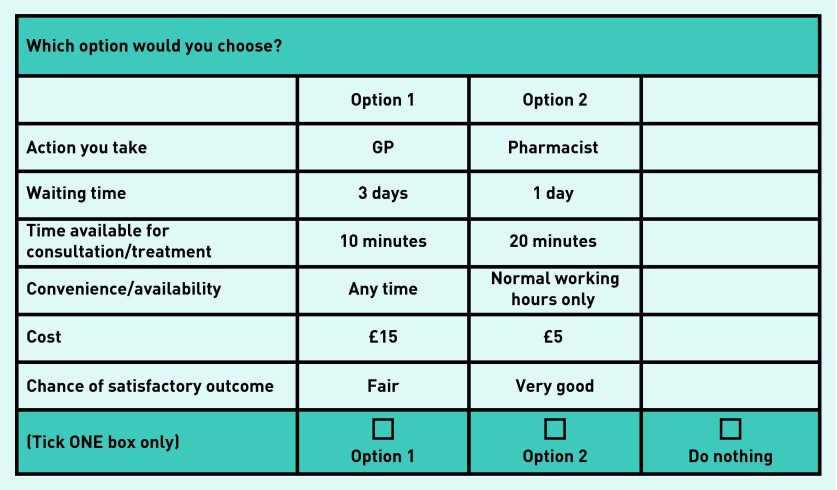
***Example choice question.***

Forty-eight choices per symptom were selected using SAS design software (maximizing D-efficiency) with restrictions placed on combinations of ‘waiting time’ and ‘action you take’ to ensure realistic choices. To reduce responder burden, the 48 choices were split into six sets of eight choices. Responders completed eight choices for each symptom. Each choice included two management options and a ‘do nothing’ alternative (Figure 1). For each symptom, responders were asked their chance of a satisfactory outcome if they chose to do nothing. To control for ordering effects, six versions of each questionnaire were developed, covering all order permutations for the three symptoms.

### Sample

The sample frame was the 1370 individuals who completed the community-based symptom survey and agreed to participate in further research. The sample for the original community-based study was an age- and sex-stratified random sample of 8000 working age adults (aged 18–60 years) drawn from 20 general medical practices across the UK (including Scotland, England, and Wales). Practices were recruited from the nationally representative Medical Research Council General Practice Research Framework, and varied in terms of their size, geographical location, rural/ urban nature, and level of deprivation. Individuals sampled were randomly assigned to one of the 36 questionnaire versions. The method of administration was a self-completion postal questionnaire. After 3 weeks non-responders were sent a reminder and replacement questionnaire.

### Statistical analysis

Equation 1 below was estimated using an alternative-specific multinomial probit regression:

$$\begin{array}{ll} V_{ij} & =\beta_{0j}\\ & +\beta_{1}{\mathrm{ActSelf}}_{j}+\beta_{2}{\mathrm{ActNHS}}_{j}+\beta_{3}{\mathrm{ActNur}}_{j}+\\ & \beta_{4}\mathrm{ActPhar}+\beta_{5}{\mathrm{ActComp}}_{j}+\beta_{6}{\mathrm{ActGP}}_{j}\\ & +\beta_{7}{\mathrm{WaitTime}0h}_{j}+\beta_{8}{\mathrm{WaitTime}1h}_{j}+\\ & +\beta_{9}{\mathrm{WaitTime}6h}_{j}+\beta_{10}{\mathrm{WaitTime}1d}_{j}+\\ & \beta_{11}{\mathrm{WaitTime}3d}_{j}+\beta_{12}{\mathrm{WaitTime}8d}_{j}\\ & +\beta_{13}\mathrm{Consultation}\;{\mathrm{Time}}_{j}\\ & +\beta_{14}{\mathrm{ConvWork}}_{j}+\beta_{15}{\mathrm{ConvAny}}_{j}\\ & +\beta_{16}{\mathrm{OutcomePoor}}_{j}+\beta_{17}{\mathrm{OutcomeFair}}_{j}+\\ & \beta_{18}{\mathrm{OutcomeGood}}_{j}\\ & +\beta_{19}{\mathrm{OutcomeVeryGood}}_{j}+\beta_{20}{\mathrm{Cost}}_{j} \end{array}$$

Utility (benefit) from the management regimen is represented by *V*, characterised by different combinations of attribute levels. Subscript *i* denotes the individual and *j* the alternatives within a choice set. *β_0j_* is the alternative-specific constant, showing the general preference to do something rather than nothing; a positive *β_0_* implies a general preference to do something. Effects coding was used for ‘action you take’, ‘convenience/availability’, ‘chance of satisfactory outcome’, and ‘waiting time’. ‘Consultation time’ and ‘cost’ were modelled as continuous variables. Equation 1 was estimated for all individuals who completed at least one choice and provided information on their perceived chance of a satisfactory outcome if they chose ‘do nothing’.

From Equation 1, WTP for a marginal change in an attribute is estimated as $-\left(\frac{\beta_{i}}{\beta_{\mathrm{cost}}}\right)$

Thus, for example, $-\frac{\beta_{6}}{\beta_{20}}$ indicates WTP to see a GP and $-\frac{\beta_{14}}{\beta_{20}}$ WTP for normal working hours only. If positive, $-\left(\frac{\beta_{0}}{\beta_{\mathrm{cost}}}\right)$ indicates a preference to do something (over nothing). WTP was estimated for a marginal change in all statistically significant attributes, with confidence intervals (CIs) obtained using the delta method. Separate models were estimated for each symptom as well as sex, age, and household income. The likelihood ratio test was used to test if preferences differed across subgroups.

## RESULTS

Of 1370 questionnaires, 851 were returned completed, 61 blank, 18 were undelivered, and three recipients were unable to complete, giving a corrected response rate of 63.1%. Appendix 3 shows responder characteristics and comparable UK figures. Responders were more likely than national figures to be female, older, married, better educated, and have a higher socioeconomic status. As intended, responders rated diarrhoea as less serious (mean score 2.9, standard deviation [SD] 1.99) than dizziness (mean score 5.22, SD 2.09) or chest pain (mean score 8.65, SD 1.65).

Over 98% of responders completed at least one DCE choice per symptom, with 95% of those completing all eight. Approximately 5% did not provide information on satisfactory outcome when doing nothing. This resulted in 795 individuals included in the analyses for diarrhoea, 797 for dizziness, and 793 for chest pain. Alternative-specific multinomial probit regression models, WTP values, and CIs for each symptom are presented in Table 1. The likelihood ratio (LR) test rejected the null hypothesis that preferences were the same between symptoms (LR 1288.86 ∼ χ^2^(degrees of freedom [df] 13)).

**Table 1.** Results of alternative-specific multinomial probit regression analyses and WTP values

		**Diarrhoea**	**Dizziness**	**Chest pain**
**Constant**	**Constant (*β*)**	0.031	0.4686^b^	1.0552^b^
	WTP (95% CI)	n/s	£25.12 (£18.76 to £31.47)	£160.47 (£111.06 to £209.9)

**Action you take**	**Self-care (*β*)^a^**	0.6811^b^	−0.0994^b^	−0.4807^b^
	WTP (95% CI)	£32.61 (£27.62 to £37.61)	−£5.33 (−£10.03 to £0.63)	−£73.11 (−£86.95 to −£59.26)

	**NHS 24/NHS Direct (*β*)**	−0.1620^b^	0.0017	0.0610^b^
	WTP (95% CI)	−£7.76 (−£12.09 to −£3.42)	n/s	£9.27 (£0.47 to £18.07)

	**Nurse (*β*)**	−0.0367	0.3381^b^	0.2779^b^
	WTP (95% CI)	n/s	£18.12 (£13.04 to £23.20)	£42.26 (£31.24 to £53.28)

	**Pharmacist (*β*)**	0.2922^b^	−0.0007	−0.0943^b^
	WTP (95% CI)	£13.99 (£9.51 to £18.48)	n/s	−£14.34 (−£24.33 to −£4.35)

	**Complementary practitioner (*β*)**	−0.6799^b^	−0.648^b^	−0.3012^b^
	WTP (95% CI)	−£32.56 (−£37.90 to −£27.21)	−£34.73 (−£40.12 to −£29.34)	−£45.8 (−£57.22 to −£34.38)

	**GP (*β*)**	−0.0948^b^	0.4084^b^	0.5374^b^
	WTP (95% CI)	−£4.54 (−£8.94 to −£0.14)	£21.89 (£17.32 to £26.45)	£81.72 (£66.54 to £96.91)

**Waiting time**	**0 hours (*β*)^a^**	0.4620^b^	0.3905^b^	0.3799^b^
	WTP/hour (95% CI)	£22.13 (£16.71 to £27.54)	£20.93 (£15.20 to £26.66)	£57.76 (£43.86 to £71.67)

	**1 hour (*β*)**	0.3530^b^	0.3996^b^	0.4123^b^
	WTP/hour (95% CI)	£16.90 (£12.74 to £21.06)	£21.42 (£17.15 to £25.68)	£62.71 (£50.77 to £74.64)

	**6 hours (*β*)**	0.1686^b^	0.2153^b^	0.3020^b^
	WTP/hour (95% CI)	£8.07 (£4.17 to £11.98)	£11.54 (£7.47 to £15.61)	£45.91 (£35.28 to £56.54)

	**1 day/24 hours (*β*)**	0.2332^b^	0.3155^b^	0.2077^b^
	WTP/hour (95% CI)	£11.17 (£7.03 to £15.30)	£16.91 (£12.48 to £21.34)	£31.58 (£21.89 to £41.27)

	**3 days/72 hours (*β*)**	−0.1721^b^	−0.2619^b^	−0.3407^b^
	WTP/hour (95% CI)	−£8.24 (−£13.04 to −£3.44)	−£14.04 (−£18.88 to −£9.19)	−£51.81(−£63.82 to −£39.79)

	**8 days/192 hours (*β*)**	−1.0447^b^	−1.0589^b^	−0.9611^b^
	WTP/hour (95% CI)	−£50.03 (−£56.62 to −£43.44)	−£56.75 (−£63.38 to −£50.14)	−£146.16 (−£170.1 to −£122.22)

**Time available for consultation**	**Minutes, (*β*)**	−0.0001	0.0006	−0.0005
	WTP/minute (95% CI)	n/s	n/s	n/s

**Convenience/availability**	**Working hours only (*β*)^a^**	−0.0292	−0.0496^b^	−0.0193^b^
	WTP (95% CI)	n/s	−£2.66 (−£4.39 to £0.92)	−£2.94 (−£6.26 to £0.37)

	**Any time (*β*)**	0.0292	0.0496^b^	0.0193^b^
	WTP (95% CI)	n/s	£2.66 (£0.92 to £4.39)	£2.94 (−£0.37 to £6.26)

**Chance of a satisfactory outcome**	**Poor (*β*)^a^**	−0.8612^b^	−0.9646^b^	−0.6448^b^
	WTP (95% CI)	−£41.24 (−£45.62 to −£36.86)	−£51.70 (−£56.54 to −£46.86)	−£98.06 (−£114.09 to −£82.03)

	**Fair (*β*)**	−0.2389^b^	−0.2075^b^	−0.0814^b^
	WTP (95% CI)	−£11.44 (−£14.14 to −£8.74)	−£11.12 (−£13.81 to −£8.43)	−£12.38 (−£18.81 to −£5.96)

	**Good (*β*)**	0.3867^b^	0.5421^b^	0.2763^b^
	WTP (95% CI)	£18.51 (£15.77 to £21.26)	£29.06 (£25.45 to £32.67)	£42.01 (£32.83 to £51.19)

	**Very good (*β*)**	0.7134^b^	0.6299^b^	0.4500^b^
	WTP (95% CI)	£34.16 (£30.61 to £37.72)	£33.77 (£29.78 to £37.75)	£68.43 (£56.35 to £80.51)

**Cost**	**Cost (*β*)**	−0.0209^b^	−0.0187^b^	−0.0066^b^
	95% CI	−0.0228 to −0.0189	−0.0206 to −0.0167	−0.0079 to −0.0052
	*P*-value	<0.001	<0.001	<0.001

Log likelihood		−5209.892	−4877.9286	−4229.0126

Number of individuals (observations)		795 (18 417)	797 (18 507)	793 (18 450)

n/s = not significant. WTP = willingness to pay. Effects coding was used for ‘action you take’, ‘convenience/availability’, ‘chance of a satisfactory outcome’, and ‘waiting time’. ‘Consultation time’ and ‘cost’ were modelled as continuous variables.

a Using effects coding L-1 levels are calculated using the regression model, the missing level is obtained from the negative of the sum of all other coefficients.

b Significant at the 5% level.

For dizziness and chest pain the constant term was positive and significant, indicating responders prefer (all other things equal) to do something rather than nothing. For all symptoms the coefficient for cost was negative and significant, indicating that cost played an important role in responders’ preferences and that responders preferred to pay less. Responders’ WTP to manage their symptoms varied from £25.12 (95% CI = £18.76 to £31.47) for dizziness to £160.47 (95% CI = £111.06 to £209.90) for chest pain.

**Figure 2.**
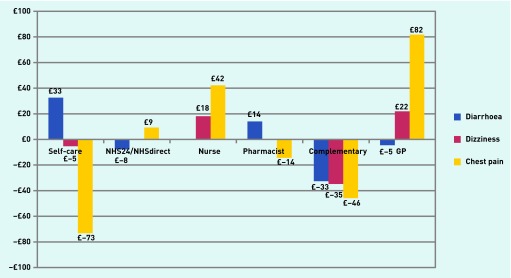
***Willingness-to-pay estimates for different management actions for all three symptoms. WTP bars are shown only for actions that contributed significantly to preferences.***

The importance of different management actions varied between symptoms (Table 1, Figure 2). Self-care was the most preferred action for diarrhoea (£32.61, 95% CI = £27.62 to £37.61) followed by pharmacist (£13.99, 95% CI = £9.51 to £18.48). There were significant preferences not to consult the GP or use NHS24/NHS Direct (indicated by the negative WTP). Practice nurse was not a significant driver of preferences for the management of diarrhoea. For both dizziness and chest pain, the most preferred action was to consult the GP. Responders valued consulting a GP significantly more for chest pain (£81.72, 95% CI = £66.54 to £96.91) than dizziness (£21.89, 95% CI = £17.32 to £26.45). Consulting a practice nurse was the next preferred action for both symptoms and there was a significant preference not to use self-care. For chest pain, the third preferred action was to use NHS24/NHS Direct (£9.27, 95% CI = £0.47 to £18.07) and there was a preference not to use the pharmacist (−£14.34, 95% CI = −£24.33 to −£4.35); neither of these actions contributed significantly to preferences related to dizziness. For all symptoms there was a preference not to use a complementary practitioner.

For all symptoms waiting time was a significant determinant of responders’ preferences, with responders preferring to wait less time, although when faced with 6 hours waiting time responders preferred a next-day appointment. WTP for a waiting time of 1 hour was largest for chest pain (£62.71, 95% CI = £50.77 to £74.64) compared with dizziness (£21.42, 95% CI = £17.15 to £25.68) and diarrhoea (£16.90, 95% CI = £12.74 to £21.06). Similarly, for all symptoms responders preferred a good or very good chance of a satisfactory outcome, and preferred not to have a poor or fair chance of a satisfactory outcome. Very good chance of a satisfactory outcome was valued significantly more for chest pain (£68.43, 95% CI = £56.35 to £80.51) than for dizziness (£33.77, 95% CI = £29.78 to £37.75) or diarrhoea (£34.16, 95% CI = £30.61 to £37.72). The significance of convenience/availability differed between symptoms. This attribute was not significant for diarrhoea, but was significant for dizziness and chest pain with responders being willing to pay £2.66 (95% CI = −£4.39 to £0.92) and £2.94 (95% CI = −£6.26 to £0.37), respectively, to manage their symptom at any time rather than during working hours only. Time available for consultation did not influence preference for any symptom.

WTP for dizziness management that involved consulting a GP, waiting 1 hour, being available at any time and having a very good outcome is:

$$\text{WTP}=£25.12_{\text{constant}}+£21.89_{\text{GP}}+£21.41_{\text{wait}1\text{hour}}+£2.66_{\text{convenience}}+£33.77_{\text{outcome}}=£104.86.$$

**Figure 3.**
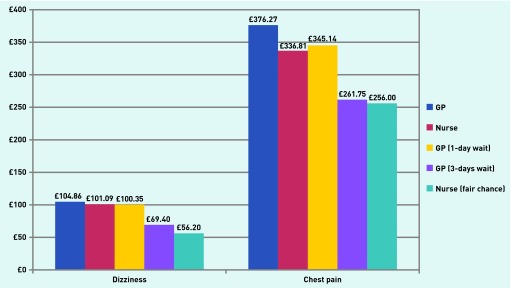
***Total willingness-to-pay estimates for alternative models of care (1-hour waiting time, available anytime, and with a very good chance of a satisfactory outcome unless otherwise stated) for dizziness and chest pain.***

Increasing waiting time by 23 hours (that is from 1 hour to 1 day) for a GP appointment reduces WTP by £4.51 to £100.35, making a 1-hour wait for the practice nurse (WTP £18.12), assuming all else is unchanged, slightly more valued (£101.09). Thus, while seeing a GP was preferred to a practice nurse, a shorter waiting time would compensate for the less preferred option. For chest pain, when faced with a 1-hour waiting time, responders value seeing a GP more than a practice nurse (WTP £376.27 versus £336.81) and are willing to wait 3 days (WTP £261.75) to see a GP if the chance of a satisfactory outcome when seeing a nurse reduces from very good to fair (WTP £256.00) (Figure 3).

Findings from likelihood ratio tests, used to test if preferences differed across subgroups, indicated that household income and age were significantly associated with preferences for all symptoms. Sex was significantly associated only with preferences for diarrhoea. WTP estimates indicated, however, that preferences for action you take within the subgroups followed the same pattern as for the whole sample (for example, self-care for diarrhoea and GP for dizziness and chest pain) (Table 2). Where differences existed, these related to the relative strength of preference (that is, one group valuing a particular preference more than another) or variations in preferences for other attribute or levels (for example, for diarrhoea, pharmacist was significant for females but not for males). Further subgroup results are available on request from the authors.

**Table 2.** Summary of WTP estimates (£) for ‘action you take’ for sex, age, and income

		**Diarrhoea**	**Dizziness**	**Chest pain**
**Sex**	**Self-care**			
	Male	29.00	−9.78	−68.83
	Female	34.76	n/s	−75.53

	**NHS 24/NHS Direct**			
	Male	n/s	n/s	n/s
	Female	−10.02	n/s	n/s

	**Nurse**			
	Male	n/s	16.11	40.90
	Female	n/s	19.17	42.89

	**Pharmacist**			
	Male	n/s	n/s	−22.79
	Female	17.73	n/s	n/s

	**Complementary practitioner**			
	Male	−32.78	−35.02	−42.92
	Female	−32.21	−34.61	−47.34

	**GP**			
	Male	n/s	24.80	81.63
	Female	−8.01	20.18	82.14

**Log likelihood**		25.054 (df = 13), *P*<0.01	n/s	n/s

**Age group, years**	**Self–care**			
	18–24	n/s	n/s	−56.15
	25–34	30.13	n/s	−72.28
	35–44	35.82	n/s	−59.04
	45–54	32.89	n/s	−71.26
	55–60	33.77	−11.87	−95.58

	**NHS 24/NHS Direct**			
	18–24	n/s	n/s	n/s
	25–34	n/s	n/s	n/s
	35–44	n/s	n/s	n/s
	45–54	n/s	n/s	n/s
	55–60	n/s	n/s	n/s

	**Nurse**			
	18–24	n/s	n/s	66.44
	25–34	n/s	n/s	42.31
	35–44	n/s	15.03	42.50
	45–54	n/s	21.96	42.09
	55–60	n/s	18.12	40.14

	**Pharmacist**			
	18–24	n/s	n/s	−63.55
	25–34	n/s	n/s	n/s
	35–44	17.85	n/s	−26.21
	45–54	15.75	n/s	n/s
	55–60	10.99	n/s	n/s

	**Complementary practitioner**			
	18–24	n/s	n/s	n/s
	25–34	−33.17	−47.77	−53.90
	35–44	−29.34	−31.80	−40.17
	45–54	−35.98	−35.44	−47.84
	55–60	−32.91	−36.75	−57.73

	**GP**			
	18–24	n/s	n/s	64.21
	25–34	n/s	19.93	70.64
	35–44	n/s	n/s	67.67
	45–54	n/s	21.39	75.87
	55–60	n/s	35.58	114.28

**Log likelihood**		26.811 (df = 13) *P*<0.01	39.918 (df = 13) *P*<0.01	51.591 (df = 13) *P*<0.01

**Annual household income, £**	**Self-care**			
	<15 000	18.98	−28.46	−88.51
	15 000–29 999	28.41	n/s	−69.86
	30 000–49 999	30.77	n/s	−71.76
	≥50 000	40.50	n/s	−67.33

	**NHS 24/NHS Direct**			
	<15 000	n/s	n/s	n/s
	15 000–29 999	n/s	n/s	n/s
	30 000–49 999	n/s	n/s	n/s
	≥50 000	n/s	n/s	n/s

	**Nurse**			
	<15 000	n/s	28.99	56.29
	15 000–29 999	n/s	24.52	40.85
	30 000–49 999	n/s	14.56	32.80
	≥50 000	n/s	15.37	43.79

	**Pharmacist**			
	<15 000	n/s	n/s	n/s
	15 000–29 999	12.50	n/s	n/s
	30 000–49 999	14.63	n/s	n/s
	>50 000	13.92	n/s	n/s

	**Complementary practitioner**			
	<15 000	−24.31	−28.95	n/s
	15 000–29 999	−24.29	−31.40	−51.06
	30 000–49 999	−34.88	−39.05	−43.63
	≥50 000	−39.31	−36.73	−57.16

	**GP**			
	<15 000	n/s	35.31	77.50
	15 000–29 999	−11.81	16.39	82.25
	30 000–49 999	n/s	22.24	85.44
	≥50 000	n/s	18.96	79.38

**Log likelihood**		872.66 (df = 13) *P*<0.01	851.29 (df = 13) *P*<0.01	790.60 (df = 13) *P*<0.01

df = degrees of freedom. n/s = not significant. WTP = willingness to pay.

## DISCUSSION

### Summary

Responders’ management preferences varied between symptoms. Self-care was the preferred option for diarrhoea, and consulting a GP the preferred option for dizziness and chest pain, with this preference being valued significantly more for chest pain. For all symptoms, there was a preference for shorter waiting times, lower cost, and a good or very good chance of a satisfactory outcome. Preferences were stronger for more serious symptoms. Greater convenience/availability was important for dizziness and chest pain, but time available for consultation did not contribute significantly to preferences for any symptom. Preference patterns were consistent across different population subgroups, although there were differences in relative strength of preferences.

### Strengths and limitations

This DCE explored preferences for the management of three symptoms within the same population at the same time. Therefore, differences found related to the symptom scenarios, not different populations studied. This DCE included a broader range of attributes than has been used in similar DCEs. Uniquely, it also explored preferences in different population subgroups.

At 63.1% the completion rate was fairly high compared with other population-based DCEs.^9^,^10^ This may be because the DCE was sent to people who had already taken part in a symptom survey and who had indicated a willingness to participate in further research. Using the same population for the DCE that had participated in earlier phases of the research may have introduced selection bias, although its impact is difficult to ascertain. For example, those responding to both questionnaires may be more interested in health-related matters and more inclined to respond in a particular way. Recruitment of practices from a wide variety of geographical and socioeconomic areas ensured that most population subgroups were well represented, thus providing a good level of generalisability. Despite this, the present sample did differed from the national UK demographic profile in a number of ways. The finding, that in most cases, differences across population groups related to the relative strength of preference rather than different management preferences *per se*, suggests that the under-representation of some population groups in this study will not have affected materially the management preferences found.

### Comparison with existing literature

Few DCEs have examined preferences for symptom management. The preference for self-care for diarrhoea is consistent with a study exploring preferences for self-care or professional advice for minor illness (flu-like symptoms).^9^ The preference for consulting a GP for potentially more serious symptoms is also consistent with a follow-up study looking at preferences for managing symptoms of differing severity,^11^ while preference for GP rather than other health professionals has been noted in previous studies.^12^–^14^ As with the present study, waiting time has been identified as important in other DCEs,^9^,^10^,^12^,^15^ although one study has suggested that the waiting time to see a GP is of limited importance.^16^ The inclusion of chance of a satisfactory outcome adds to previously reported findings, however, showing that changes in waiting time may not be sufficient to change behaviour if perceived chance of a satisfactory outcome is less favourable.

### Implications for research and practice

Despite an increased range of primary healthcare services in the UK, the present findings suggest that the traditional approaches of self-care for minor symptoms and GP for more serious symptoms remain central to preferences when managing symptoms.

Although it is important not to discourage GP consultations when necessary, other options, such as self-care or the use of other primary care health providers, should be encouraged when appropriate.^4^,^17^ The present DCE identified a number of important factors when people make management choices, and explored where people would or, equally importantly, would not be willing to trade when considering different symptom scenarios. For example, whereas previous studies have reported that patients prefer longer consultation times,^18^ the present finding that time available for consultation did not affect preferences for the three symptoms suggests that longer consultation time by itself is unlikely to influence decisions about symptom management.

Interestingly, the present study found that the relative importance of attributes varied between symptoms. While waiting time and chance of a satisfactory outcome were important for all three symptoms, their relative value changed. In general, people were more prepared to wait longer for their preferred action, and less prepared to trade a good or very good chance of a satisfactory outcome, for symptoms rated as more serious. Although tipping points for management preferences will vary among individuals and by symptom experience, understanding the way these relative values change is important as it may help to inform the development of interventions aimed at changing consultation behaviour or improving services. For example, always offering shorter waiting times for the practice nurse compared with the GP may encourage those who feel their symptom is fairly minor to see the nurse, whereas those who feel their symptom is more serious would choose to wait longer to see the GP.

The strong preference for a good or very good chance of a satisfactory outcome indicates that people will act in a way that they think will most likely achieve this. Exploring beliefs about the chance of a satisfactory outcome from different primary care services for different symptoms is an important area for future research (and education) and needs further investigation.

The continued preference for consulting a GP could indicate resistance to use other primary care professionals or services for managing symptoms. The present study has shown, however, that people are willing to trade between different health professionals, particularly for less serious symptoms. Attributes likely to affect decision making were also identified. Further research using different symptom scenarios and including other attributes could provide a more comprehensive picture.

## Appendix 1. Attributes and levels used in the discrete choice experiment

**Table:**

**Attributes**	**Levels**	**Variable in Equation (1)**	**Regression coding**
**Action you take**	Self-care	ActSelf	*β_1_*
	Practice nurse	ActNur	*β_2_*
	NHS24/NHS Direct	ActNHS	*β_3_*
	Pharmacist	ActPhar	*β_4_*
	Complementary practitioner	ActComp	*β_5_*
	GP	ActGP	*β_6_*

**Waiting time**	0 hours	WaitTime0h	*β_7_*
	1 hour	WaitTime1h	*β_8_*
	6 hours	WaitTime6h	*β*_*9*_
	1 day	WaitTime1d	*β*_*10*_
	3 days	WaitTime3d	*β_11_*
	8 days	WaitTime8d	*β_12_*

**Time available for consultation/treatment**	5 minutes		
	10 minutes	Consultation	*β_13_*
	20 minutes	Time	
	30 minutes		

**Convenience/availability**	Normal working hours only	ConvWork	*β_14_*
	Normal working hours and out of hours (evenings and weekends)	ConvAny	*β*_*15*_

**Chance of a satisfactory outcome**	Poor chance	OutcomePoor	*β_16_*
	Fair chance	OutcomeFair	*β_17_*
	Good chance	OutcomeGood	*β_18_*
	Very good chance	OutcomeVeryGood	*β_19_*

**Cost, £**	1.0		
	3.0		
	5.0		
	7.5	Cost	*β_20_*
	15.0		
	25.0		
	40.0		
	75.0		

## Appendix 2. Three symptom scenarios of increasing seriousness

**Table:**

**SCENARIO A**
**Please imagine this situation:**
*You have diarrhoea. It started mid-morning yesterday and soon you were on the toilet every hour for most of the day. You had some stomach discomfort and a feeling of nausea, but you were not sick. You appetite isn’t good, but you are managing to drink lots of fluid. A few people at work have had similar symptoms. Today (it’s now lunchtime) you have had diarrhoea a couple of times this morning.*

**SCENARIO B**
**Please imagine this situation:**
*When you got up this morning you felt dizzy. The room seemed to be spinning round about you and you had to sit back down on the bed and hold on. If you sit or lie still — avoiding any movement of your head — you feel OK. But when you get up or walk everything starts to spin again, and you feel a bit sick.*

**SCENARIO C**
**Please imagine this situation:**
*You woke up early with a discomfort across the front of your chest. It was not so much a pain as a heavy feeling — constricting. You didn’t feel right — a bit short of breath and sweaty — so you sat up on the side of your bed and opened the window for some air. The discomfort has now turned into a pain across your chest, not sharp, but dull and heavy, like someone standing on your chest and you have started to feel very nauseous.*

## Appendix 3. Participant characteristics of DCE sample and UK demographics

**Table:**

	**n^a^**	**Symptom survey sample, %**	**UK demographics, %**
**Sex**			
Male	308	36.1	49.9^b,c^
Female	542	63.9	50.1^b,c^

**Age group, years**			
18–24	41	4.9	16.5^b,c^
25–34	103	12.1	22.2^b,c^
35–44	204	24.0	25.8^b,c^
45–54	282	33.1	23.0^b,c^
55–60	220	25.9	12.5^b,c^

**Marital status**			
Single	112	13.3	42.8^b,c^
Married/living together	664	78.4	46.2^b,c^
No longer married	69	8.2	11.0^b,c^

**Social support**			
Low	34	4.1	n/a
Medium	285	34.0	n/a
High	510	61.9	n/a

**Educational status**			
No formal education	74	8.9	n/a
Secondary school or equivalent	347	41.7	n/a
Higher education	411	49.3	n/a

**Housing tenure**			
Owned/mortgaged	726	86.3	68.4^b^
Council/housing association rented	70	8.4	16.8^b^
Privately rented and other	45	5.3	14.8^b^

**Employment status**			
Full-time	436	51.7	49.7^b^
Part-time	154	18.4	15.9^b^
Self-employed	70	8.3	8.6^b^
Unable to work	40	4.7	21.4
Others not in paid employment	140	16.8	economically inactive^b^

**Annual household income, £**			
<15 000	91	11.7	∼20.0^b,d^
15 000–29 999	201	25.6	∼30.0^b,d^
30 000–49 999	249	32.1	∼40.0^b,d^
≥50 000	239	30.6	∼10.0^b,d^

**Ethnic group**			
White	820	97.9	92.1^b^
Other	18	2.1	7.9^b^

**Smoking status**			
Never smoked	484	57.5	53.0^e^
Ex-smoker	232	27.8	25.0^e^
Current smoker	123	14.7	21.0^e^

**Chronic condition**			
Yes	389	46.2	29.0^e,f^
No	452	53.8	71.0^e^

n/a = Comparable data not available.

a Total numbers for each group may not add up to full sample because of missing data in participant characteristics categories.

b Office for National Statistics data.

c Working age population specifically (18–60 years).

d Average gross income by decile groups of non-retired households.

e General Lifestyle Survey, 2008.

f Proportion of persons aged 16–64 years who reported a longstanding illness.
